# Efficacy and safety of deep brain stimulation in OCD

**DOI:** 10.1192/j.eurpsy.2023.110

**Published:** 2023-07-19

**Authors:** J. M. Menchon

**Affiliations:** Bellvitge University Hospital. University of Barcelona. Cibersam, Barcelona, Spain

## Abstract

**Abstract:**

Deep brain stimulation has become a last resource procedure for severe, chronic and refractory OCD. However, the procedure is highly invasive and its efficacy must be balanced against the risks it may entail. In addition, there are a number of procedure-related issues that may influence the efficacy of DBS, such as the target of stimulation, the stimulation parameters to be selected, the presence of comorbidities as exclusion criteria, or the efficacy in different OCD subtypes, among others. A comprehensive review of the knowledge and experience of DBS in OCD may be useful in helping to select appropriate candidates.

**Disclosure of Interest:**

None Declared

